# Supplementary material for: Phosphatidylserine-Exposing Annexin A1-Positive Extracellular Vesicles: Potential Cancer Biomarkers
Source: Vaccines (Basel). 2023 Mar 13;11(3):639. doi: 10.3390/vaccines11030639 (PMC10059271; doi:10.3390/vaccines11030639)
Supplement: Supplementary file 1 [file vaccines-11-00639-s001.zip › vaccines-2245946-supplementary.pdf]

Supplementary Material

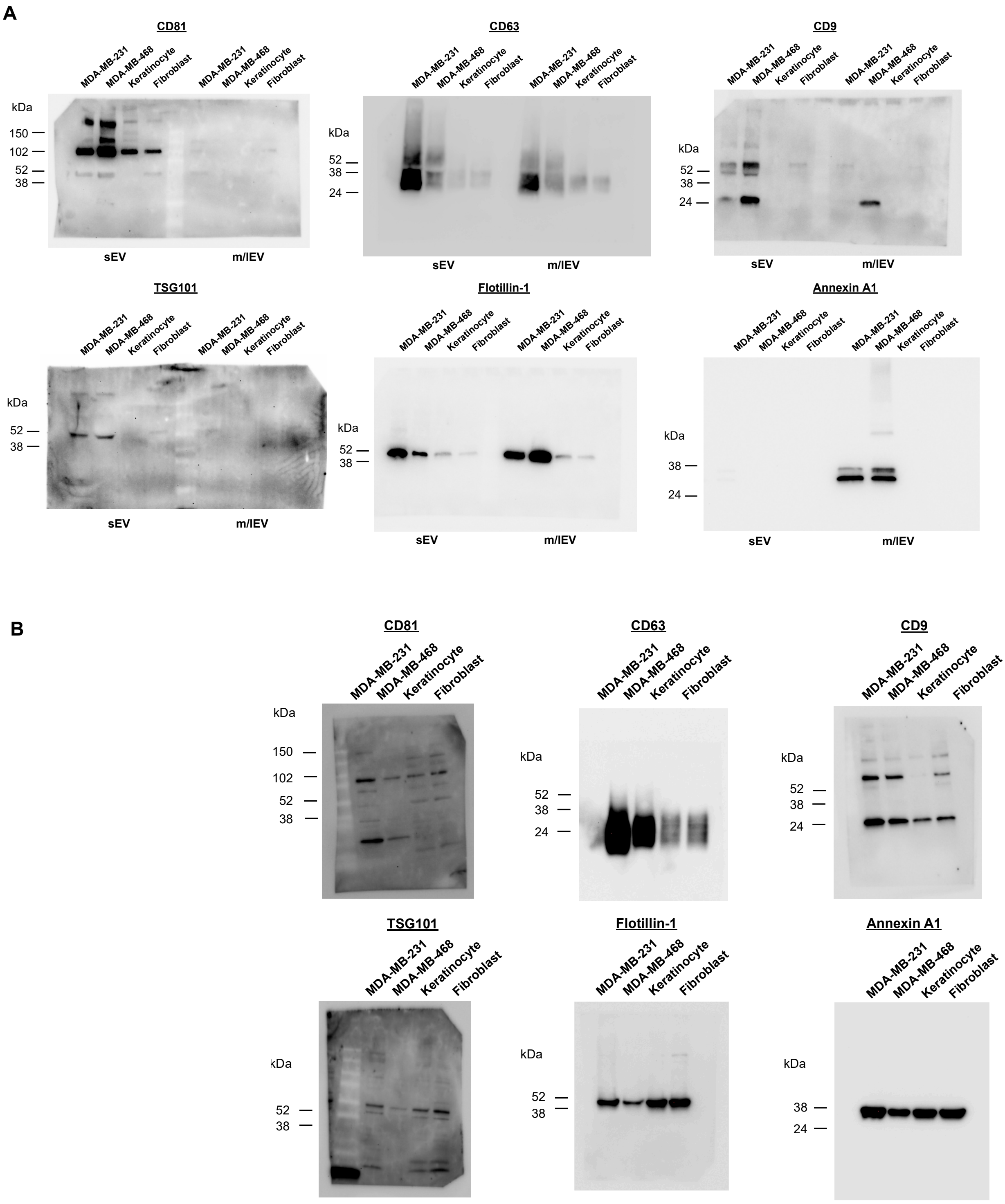

Figure S1. Western blot analysis of EV markers in sEVs and m/lEVs derived from equivalent numbers of parental cells. (A) The expression of EV markers, CD81, CD63, CD9, TSG101, flotillin-1, and annexin A1, in sEV- and m/lEV-enriched fractions. Original images for Figure 1D. (B) EV markers expressed in equal numbers of cancer (MDA-MB-231, MDA-MB-468) and non-cancerous cells (keratinocyte, fibroblast).

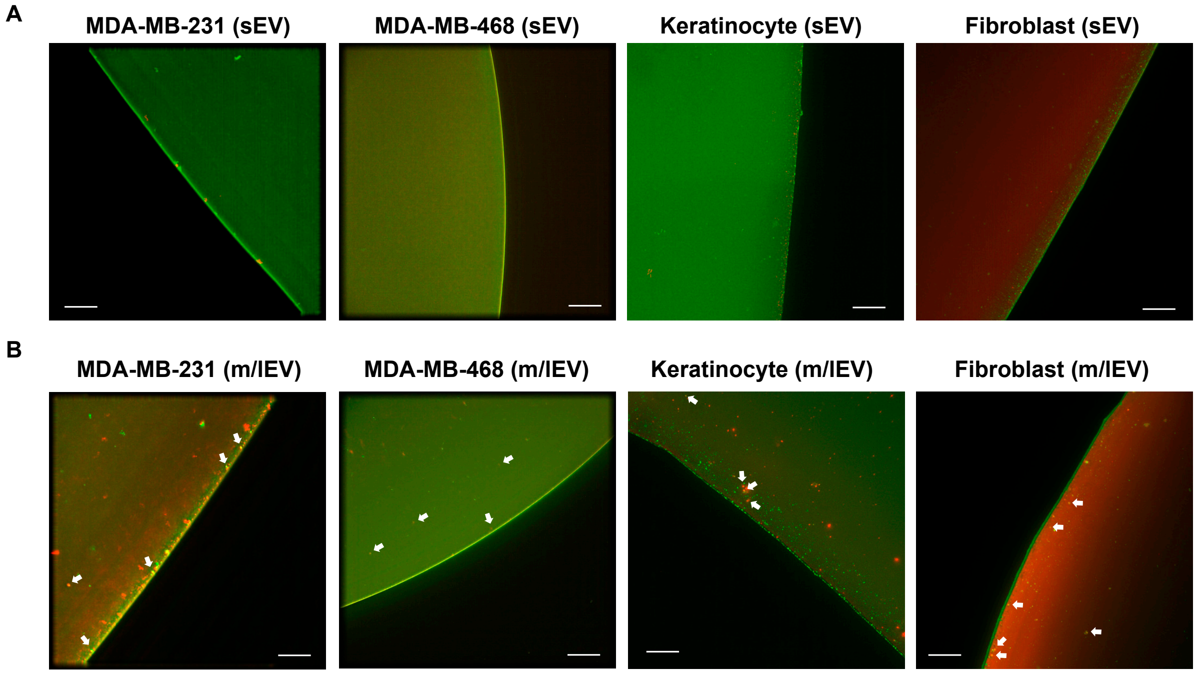

Figure S2. Recognition of EV subpopulations by FITC-GlaS and APC-annexin A5 proteins. (A and B) Dual-staining of sEV- and m/lEV-enriched fractions derived from cancer cells (MDA-MB-231 and MDA-MB-468) and non-cancerous cells (keratinocyte and fibroblast) by FITC-GlaS (green) and APC-annexin A5 (red). Arrows indicate FITC and APC double-positive fluorescent puncta. Scale bar, 10 μm.

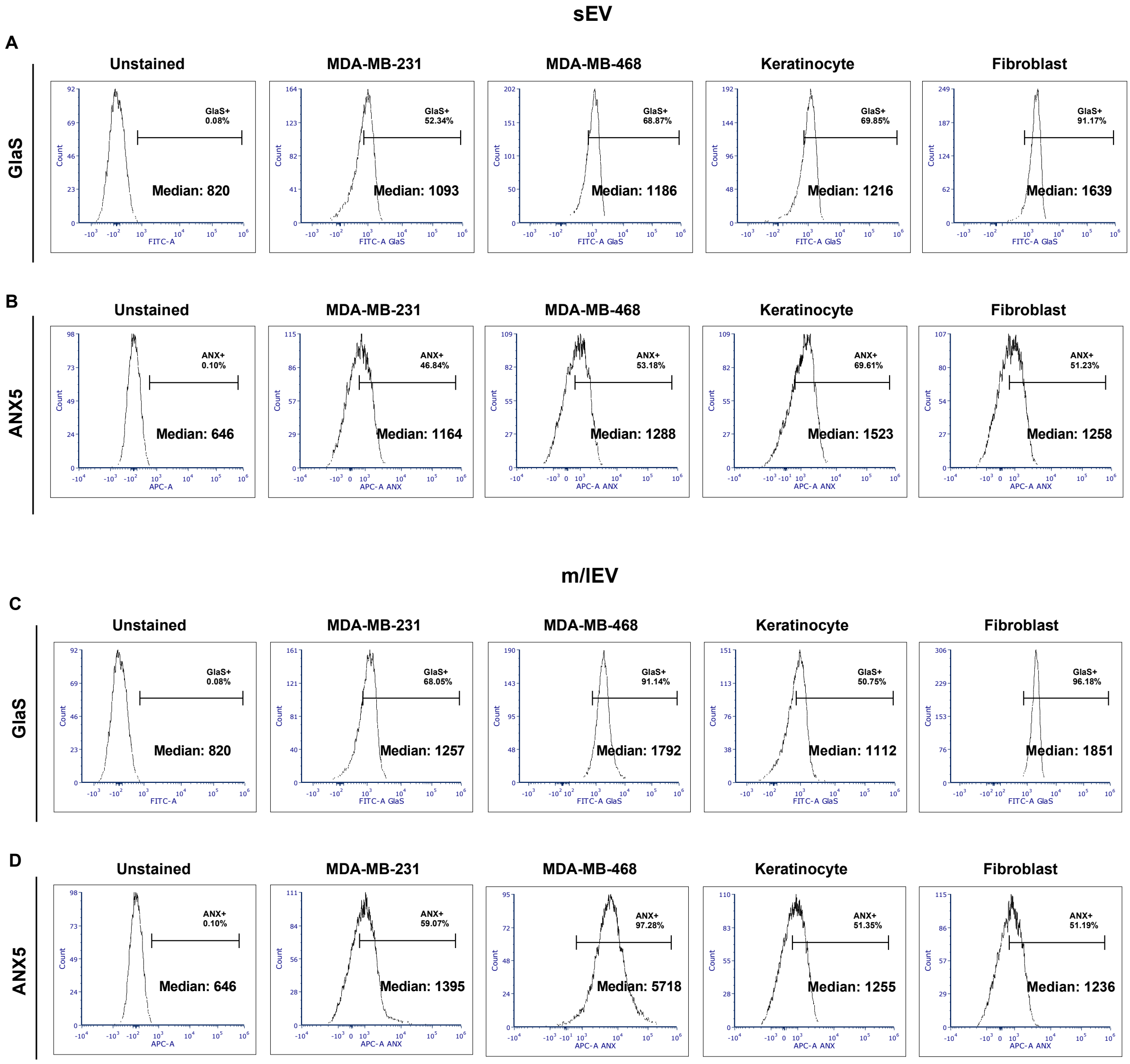

Figure S3. A representative of five analyses of PS exposure on the beads carrying sEVs or m/lEVs derived from cancer and non-cancerous cells. FITC-GlaS or APC-annexin A5 (ANX5) detected PS-exposing sEVs (A and B) or m/lEVs (C and D) on the beads. Marker gates denoting either % annexin A5+ (ANX+) or GlaS+ were set based on FMO control (unstained EV-loaded beads). Each histogram shows the median fluorescence intensity of FITC-GlaS or APC-ANX5 for singlet beads.

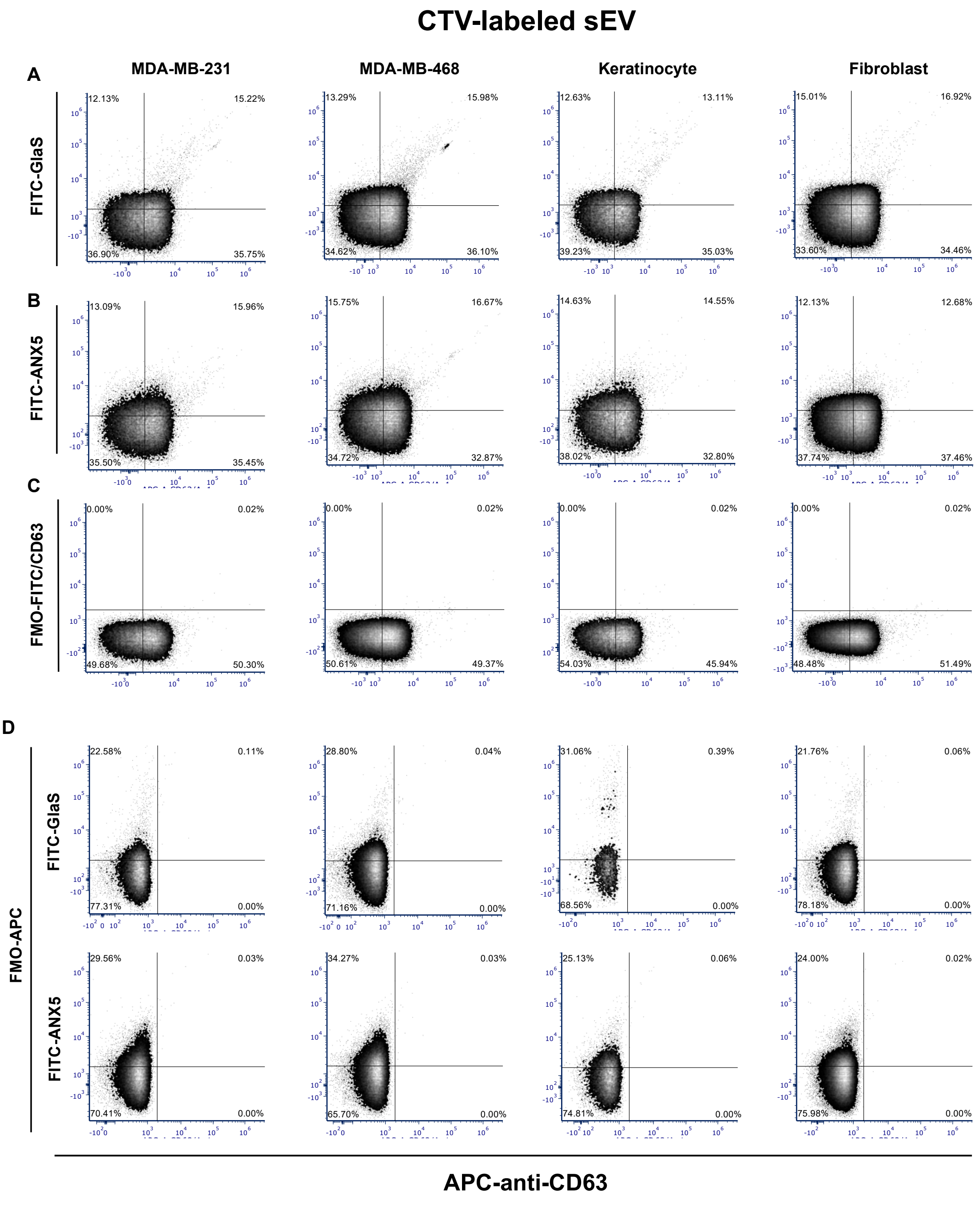

CTV-labeled sEV
A
B
C
D
MDA-MB-231
MDA-MB-468
Keratinocyte
Fibroblast
FITC-GlaS
FITC-ANX5
FMO-FITC/CD63
FMO-APC
FITC-GlaS
FITC-ANX5
APC-anti-CD63

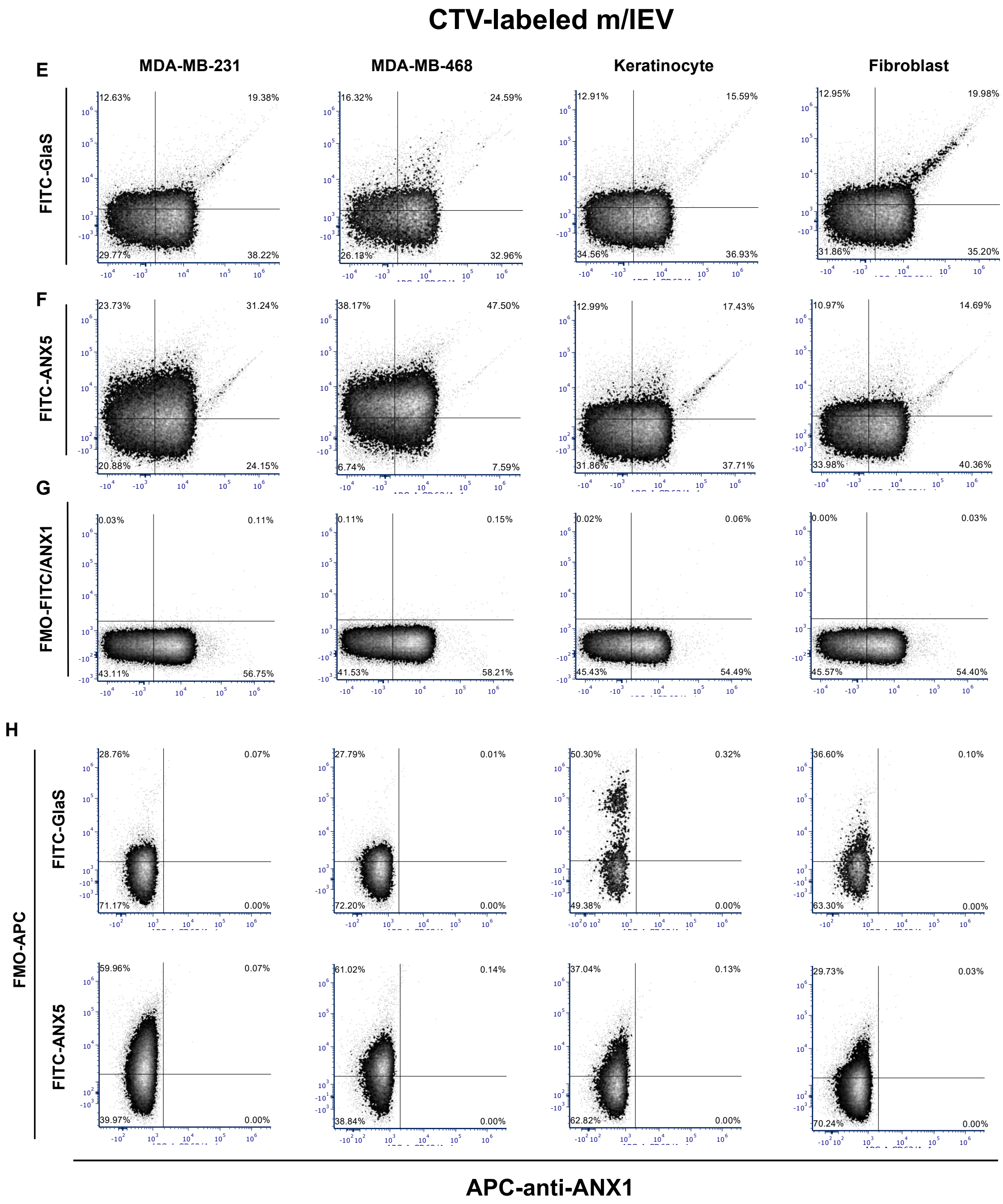

Figure S4. A representative of three independent analyses of PS-exposing sEVs and m/lEVs derived from cancer and non-cancerous cells. EVs were stained with CellTrace Violet (CTV). (A-D) CTV-sEVs were incubated with APC-anti-CD63 antibodies and FITC-GlaS or FITC-annexin A5 (ANX5). (E-H) CTV-m/lEVs were incubated with APC-anti-annexin A1 (ANX1) antibodies and FITC-GlaS or FITC-ANX5. FITC-annexin A5 (ANX5) or FITC-GlaS was evaluated to determine the frequency of individual PS-exposing EVs. Quadrant gates were set based on fluorescence minus one (FMO) controls (C, D, G, and H).
